# The opioid crisis: past, present and future policy climate in Ontario, Canada

**DOI:** 10.1186/s13011-017-0130-5

**Published:** 2017-11-02

**Authors:** Kristen A. Morin, Joseph K. Eibl, Alexandra M. Franklyn, David C. Marsh

**Affiliations:** 1 Laurentian University, Sudbury, ON Canada; 2 Northern Ontario School of Medicine, Sudbury, ON P3E 2C6 Canada; 3 Canadian Addiction Treatment Centres, Richmond Hill, ON Canada

**Keywords:** Health policy, Opioids, Coordinated care, Opioid agonist therapy, Substance use disorders, Ideology

## Abstract

**Background:**

Addressing opioid use disorder has become a priority in Ontario, Canada, because of its high economic, social and health burden. There continues to be stigma and criticism relating to opioid use disorder and treatment options. The result has been unsystematic, partial, reactive policies and programs developed based on divergent points of view. The aim of this manuscript is to describe how past and present understandings, narratives, ideologies and discourse of opioid use, have impacted policies over the course of the growing opioid crisis.

**Commentary:**

Assessing the impact of policy is complex. It involves consideration of conceptual issues of what impacts policy change. In this manuscript we argue that the development of polices and initiatives regarding opioids, opioid use disorder and opioid agonist treatment in the last decade, have been more strongly associated with the evolution of ideas, narratives and discourses rather than research relating to opioids. We formulate our argument using a framework by Sumner, Crichton, Theobald, Zulu, and Parkhurs. We use examples from the Canadian context to outline our argument such as: the anti- drug legislation from the Canadian Federal Conservative government in 2007; the removal of OxyContin™ from the drug formulary in 2012; the rapid expansion of opioid agonist treatment beginning in the early 2000s, the unilateral decision made regarding fee cuts for physicians providing opioid agonist treatment in 2015; and the most recent implementation of a narcotics monitoring system, which are all closely linked with the shifts in public opinion and discourse at the time of which these policies and programs are implemented.

**Conclusion:**

We conclude with recommendations to consider a multifactorial response using evidence and stakeholder engagement to address the opioid crisis, rather than a reactive policy approach. We suggest that researchers have an important role in shaping future policy by reframing ideas through knowledge translation, formation of values, creation of new knowledge and adding to the quality of public discourse and debate.

## Background

Opioid Use Disorder (OUD) continues to be a health concern in Canada and the United States [1–5]. The prevalence of OUD can be estimated by examining the number of individuals who seek treatment for their opioid dependence. The number of individuals enrolled in Opioid Agonist Treatment (OAT) in Ontario, Canada, has increased from 6000 patients to over 40,000 patients from the year 2000 to 2016 [6, 7]. Ontario is Canada’s largest province, with more than 13.2 million residents in 2010, all of whom have access to publicly funded health insurance for physician and hospital services.

The opioid epidemic is a complex, multicomponent issue. For example, over 50% of individuals with OUD also have a mental health disorder [8]. In fact, preliminary analysis from a large retrospecitve cohort study using administrative data, indicates that 87% of individuals with OUD in Ontario, also have a diagnosed mental health (MH) disorder (Table 1). Moreover, OUD is most prevelant in individuals between 15 and 34 (Table 1). There has been much evidence demonstrating that social determinants of health also have an important role in the prevention and treatment of OUD [9–11].

**Table 1:** Prevalence of mental health comorbidities and demographic characteristics within a cohort of individuals with opioid use

	MH dx	no MH dx	Total
N	55,416	8117	63,533
% of total	87.22%	12.78%	
Sex			
Male	20,988	1903	22,891
	37.87%	23.44%	36.03%
Female	34,428	6214	40,642
	62.13%	76.56%	63.97%
Age group			
15–34	28,273	4905	33,178
	51.02%	60.43%	
35–59	25,588	2985	28,573
	46.17%	36.77%	
60+	1555	227	1782
	2.81%	2.80%	
Rurality			
Urban residence	48,689	6745	55,434
	87.86%	83.10%	
Rural residence	6696	1367	8063
	12.08%	16.84%	
Income quintile			
1 (lowest)	19,367	2724	22,091
	34.95%	33.56%	34.77%
Northern Ontario	7767	1470	9237
	14.02%	18.11%	14.54%
HIV	569	30	599
	1.03%	0.37%	0.94%
Overall Mortality	2506	207	2713
	4.52%	2.55%	4.27%

Ontario, Canada data, derived from administrative health data gathered by the Institute of Clinical and Evaluative Sciences

Substance dependence, such as OUD, has been and continues to be highly stigmatized, has risen to national and international policy agendas. Sadly, perhaps one of the largest barriers limitting our collective ability to address the opioid crisis in Ontario, Canada, is the lack of consensus of the extent of the problem and uncoordinated ideas of appropriate solutions. For instance the perception of appropriate solutions to address the opioid crisis in the eyes of law enformcement, medicine, pharmacology, community programs, public health and health policy, are sometimes incompatible [12–16]. Further, there seems to be a discrepancy between research and policy ideas [12]. Here we highlight the importance of aligning ideological points, and public opinion with sound research findings for the successful development and implementation of policies and programs. The purpose of this commentary is to describe how past and present understandings, ideologies and discourse of OUD have been closely related to the development of policy for the growing opioid crisis. We describe this phenomena through the lens of a framewok developed by Sumner, Crichton, Theobald, Zulu, and Parkhurs 2011 [12], which describes research’s impacts on policy.

### Evidence on treatment

Opioid agonist treatment (OAT), and harm reduction, are critical parts of the strategy to address the epidemic of opioids. OAT is currently the standard of care and the intervention with the best evidence for long term patient safety, social wellness, and physical health benefits for the treatment of OUD [17]. For this reason, the World Health Organization has recognized OAT (both methadone and buprenorphine/naloxone, also known as suboxone) on their list of essential medicines [18].

Evidence to support the efficacy of OAT is well established in the academic literature. In a systematic literature review by Mattick, Been, Kimber and Davoli, 2009, methadone was statistically significantly more effective than non-pharmacological approaches in retaining patients in treatment and in the suppression of heroin use in 6 randomized control trials (RR = 0.66 95% CI 0.56–0.78). A cohort study, Ball and Ross, 1991, indicated that long term OAT was associated with a 97% decline in illicit opioid use. In the same study, 18.6% of patients reported ceasing intravenous drug use upon their admission to the OAT program. Importantly, Ball and Ross, followed patients after they dropped out of treatment and discovered that 82% of the drop out cohort had relapsed after 10 to 12 months being out of treatment [13, 14, 17, 19]. OAT treatment also has been shown to be associated with a significant reduction in overall crime, overall mortality [20, 21] and decreased infectious diseases related to injection drug use [14, 21–23]. Additionally, OAT has been established as a safe and effective prolonged treatment for OUD, compared to other treatment options [24, 25]. Importantly, Amato et al., 2005 concluded that the provision of OAT should not be abandoned in the absence of resources for additional psychosocial treatment as a result of their Cochrane review [13].

OAT remains the clinical strategy with the best evidence to support its effectiveness [18], but it is important to recognize that OAT is designed as a treatment and maintenance intervention for individuals with OUD. Within the context on the opioid crisis, social determinants of health such as: poverty, trauma, mental health and social exclusion should also be considered as underlying health problems associated with OUD [11–13]. The relationship between opioid treatment and social determinants is important to highlight for prevention and treatment purposes, and should be considered by those responsible for decision making relating to the opioid crisis.

OAT is expanding in Canada; yet, there continues to be a discrepancy between research and policy ideas [12]. There continues to be discourse in Ontario which has resulted in debates relating to the need to expand OAT [26, 27]. Without abandoning the need for health promotion and prevention initiatives, with the current state of OUD, we suggest continuing to expand OAT treatment alongside prevention initiatives as a way to mitigate the current opioid crisis. We encourage policy makers, and providers to use a comprehensive assessment of evidence to inform policy decisions relating to interventions for substance use disorder in the future.

### Evolving ideologies and policies

**Fig. 1:**
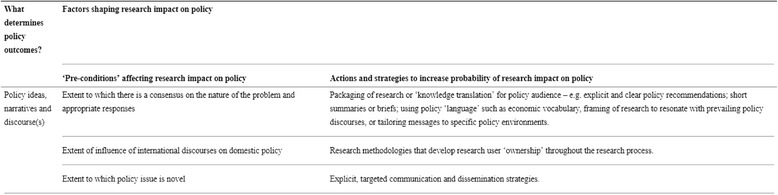
An analytical framework for factors shaping research impact on policy (Sumner, Crichton, Theobald, Zulu, Parkhurst, 2011)

In the last decade or so, public opinion of opioid use has shifted from the discussion of drug users as criminals, and the fear of narcotics [28], addiction as a character flaw [29], and most currently, that high opioid prescribing by physicians has contributed to the current opioid crisis [30–33]. Context is particularly important to understand policy ideas [12]. In the next section of this manuscript, we use the first component of the Sumner et al. framework which includes: “Policy ideas, narratives and discourse” (see Fig. 1) to describe how ideas, narratives and discourse of opioids and opioid users has shaped past and present policies in Ontario, Canada.

In 2006, under Federal legislative authority, the notion of drug users as criminals was reinforced with policies implemented by the Conservative Party of Canada throughout their 10-year term, but specifically in October of 2007 with the release of a National Anti-Drug Strategy [34]. This was a strategy which encouraged the condemning and criminalizing of drug use and drug users [34, 35]. Today, under a Liberal Government, the conversation has shifted slightly from condemning to supporting drug users, with recent initiatives such as: support for the expansion of safe injection sites and the expansion of Naloxone as a lifesaving medication for opioid-overdose [5]. Conceivably, the social construction of individuals with substance use disorders have important implications for policy outcomes [36].

In 2012, the idea that one drug was to blame for the increasing opioid issue was reinforced by the removal of OxyContin™ from the Ontario Drug Benefit Formulary 12 years after being introduced [37]. Less than 2 months after this announcement was made, all coverage for OxyContin™ ceased [37]. At that time, there was increasing attention on the liberal prescribing of OxyContin™, the escalating misuse leading to pharmacy robberies and increases in opioid dependence. In Ontario Canada, prescriptions of oxycodone increased by 850% between 1991 and 2007 [1]. The sole focus on OxyContin™ policy at the time has had serious unintended consequences. It appears that the removal of OxyContin™ drove opioid users to alternative substances once their drug of choice was no longer available [38, 39]. Rates of heroin use and Hydromorph Contin™ prescribing [40, 41] increased exponentially during that time [42].

The impacts of new policies are difficult to measure, however, with more public consultation, those involved in the decision to remove OxyContin™ may have gained insight on the potential effects of this policy on patients and health care providers. Increased services, support for primary care physicians who are typically responsible for those patients with chronic pain and, coordination of care between physicians and addiction services may have mitigated the effects of the crisis following the removal of OxyContin™. Public consultation and a critical evaluation of factors arising should be considered by those involved in planning and policy decisions for opioids in the future.

In 2012, the narcotics monitoring system was implemented in concert with the increasing discourse on physician prescribing practices in the media and in the academic literature. The system was implemented as a surveillance tool to monitor physicians’ prescribing practices [43]. With a focus on physicians, this initiative has the potential to encourage responsible prescribing practices, however other measures should also be considered to support physicians over the course of this change including: incentives to coordinate with addiction treatments, alternatives to opioid prescribing and a critical analysis of the presence, production and use of synthetic opioids.

In 2015, the Ontario government made a unilateral decision to reduce the urine drug screening billing fee as a way to mitigate the increasing costs of OAT provision in Ontario. This decision was convergent to the public discourse questioning the need for OAT and the rhetoric of physicians being financially driven to enter the practice of addiction medicine [44–46]. The urine drug screen fees in Ontario are the primary source of income which fee for service clinics use to cover overhead costs. These overhead costs are paid by each physician to operate clinics and provide infrastructure to deliver OAT across the province. With the fee cuts implemented, concern had risen because northern, rural, and remote clinics in Ontario tend to service a smaller number of patients, and the new fee structure reduces the financial viability of these clinics. This is especially important for northern, rural regions of Ontario, where more than half of patients live 126 km or more from their OAT provider; this is compared to only 16 km for patients living in Southern Ontario [47]. The number of providers able to prescribe methadone has increased over the last 10 years, and there are currently over 450 physicians in Ontario carrying a methadone exemption. Despite the expansion of OAT in Ontario, access to addiction therapy is not uniformly distributed across all regions of the province [48]. Significant needs still exist in rural and northern areas [47, 49].

Sumners et al. highlight that the dynamics of ideology and discourse can impact research uptake for decision making. In Canada, there is a lack of consensus between actors and networks of actors from very different ideological and disciplinary backgrounds relating to harm reduction and more specifically OAT. For example, although methadone maintenance treatment is considered the standard of care for OUD, methadone is one of the most highly debated, regulated, and controlled interventions in addiction treatment and one of the most regulated in medicine [50]. To prescribe methadone for analgesia or for the treatment of opioid dependence, physicians must be exempted under section 56 of the Controlled Drugs and Substances Act, require additional training, and receive regular audits on their patient files. Additionally, the current regulations and model of care for OUD in Ontario promotes access but does not incentivize efforts towards coordination with other parts of the health care system. This is important because, approximately 50% of individuals with OUD also have a concurrent mental health disorder [51]. This sub-category of mental health care recipients represents some of the most vulnerable and marginalized individuals in Ontario. Thus, there is a missed opportunity for complex patients with co-occurring mental health and OUD to receive care for their mental health and substance use disorders concurrently. We argue that this discourse and lack of consensus is linked to the state of OAT in Ontario.

### Where are we now?

Despite the efforts, initiatives and policy changes implemented in the last decade, the negative societal impact of opioids continues. With the growing availability of powerful opioids—including fentanyl and carfentanil— the crisis is becoming more obvious [52].

Although the burden of opioids on society is difficult to estimate, it is well known that the costs associated with OUD are extensive [53]. The estimated total cost in the United States of nonmedical use of prescription opioids according to Hansen et al., 2011, was $53.4 billion, of which $42 billion (79%) was attributable to lost productivity, $8.2 billion (15%) to criminal justice costs, $2.2 billion (4%) to drug abuse treatment, and $944 million to medical complications (2%) [54]. The rates of opioid-related emergency department visits and hospitalizations—both costly services in the health care system—are growing in Ontario [7, 55]. For instance, in Ontario, emergency department visits increased from 9.42 per 100,000 population in 2003 to 19.55 per 100,000 population in 2015 [55]. Social costs in the form of loss of productivity, violence, and links with crime can also be associated with substance use, and these factors can result in overwhelming economic burden, as described in the World Health Organization [56]. Additionally, OUD and substance use is often associated with poverty and social exclusion, and can lead to acute and chronic health problems [56]. OUD is often correlated with injection drug use (IDU), which is closely related to communicable diseases such as HIV, Hepatitis B, and Hepatitis C through the sharing of needles and high-risk sexual behaviors [57, 58]. Further, the most critical health issue currently facing Ontario is the threatening rise of opioid-related poisoning deaths [7, 59]. Currently, Ontario ranks second in opioid related deaths in 2016, with 865 deaths reported (5.0 to 9.9 deaths per 100,000 population). This compared to British Columbia, with 978 deaths in 2016 (20.0 and higher per 100,000 population), Alberta with 586 (10.0 to 14.9 per 100.00), and Quebec with 140 (0 to 4.9 per 100,000 population).

In the present time, there is increasing public awareness and interest around the issue of opioids. Strategies which steer away from the stigma of substance use and towards patient centered initiatives have become more common in recent years. The Ministry of Health and Long-Term Care in Ontario, has been increasing efforts to enhance patient centered care through initiatives such as the Patients First Action Plan, which was updated in 2015 [60]. In response to the overdose crisis, the distribution of Naloxone—a drug designed to reverse the effects of opioid-overdose—is starting to expand after the federal government’s announcement of the importance of this life-saving intervention. Harm reduction models, such as safe injection sites, including heroin assisted treatment (HAT), are receiving more public support [61, 62] than they have in past years [34]. For example, HAT has been recommended in the most recent Toronto Overdose Action Plan: Prevention & Response. Additionally, the Federal Minister of Health just recently approved the expansion of three safe injection sites in Montreal, Quebec and there are pending applications for another 10 sites including: three in Toronto, Ontario, two in Vancouver and Surrey, British Columbia, and one in Victoria, British Columbia and Ottawa, Ontario [63]. Lastly, there have been many policy documents recommending the integration and expansion of supports for individuals with OUD [5, 29, 64] which brings to light not only the importance of coordinated services but the advantage of aligning polarized ideas of OUD treatment. At the time of publication, government organizations were discussing progressive initiatives; however, in order to make these actionable, divergent points of view need to be unified.

### Beyond reactive policy, bringing ideas together

Some jurisdictions such as Switzerland and Vancouver, Canada have been especially successful at reframing ideas about substance use disorders and treatment. Switzerland was successful at reframing the issue of OUD in a different way. For example, such a change occurred when provision of needles to injecting drug users was reframed from a policy of drug maintenance to a policy of harm reduction for HIV [65]. Changing the discourse around an issue, may in some cases help to overcome political reluctance to target services to marginalised groups. As well, Vancouver, is a suitable example where aligned views has assisted in the progression towards improved interventions. For example, Vancouver has been especially successful at highlighting that the rising death toll related to opioids is affecting individuals across socio-economic classes [66, 67]. This type of message is a cornerstone of their progressing programing. In addition, Vancouver has been successful in brining actors together with efforts spanning across a number of different institutions (politicians, academics, community members, local businesses and law enforcement officials) to focus on a collective solution to the opioid crisis [34, 68–71]. With their collective efforts, they are often the region leading in innovative solutions such as Insite [68, 69, 71]. Insite is North America’s first legal supervised injection site, providing heroin assisted treatment for long-term drug users. Insite operates under a harm-reduction model and strives to decrease the adverse health, social and economic consequences of drug use without requiring abstinence from drug use [72].

## Conclusion

Here we demonstrated how attitudes and policy decisions are closely associated: when not aligned with evidence, policies can have unintended consequences. Vancouver and Switzerland are examples of how attitudes and patient centered planning, can influence policy decisions and entice people to come together which has the potential to create positive change. In Ontario, policies and ideological points of view are shifting into more preventative, holistic, patient centered models; however there continues to exist stigma, blame, and competing ideologies of addiction treatment. Despite efforts and evolving policies in Ontario, the missing piece to make policies actionable is the focus on aligning views and a critical evaluation of all factors impacting the issue rather than taking reactive action on the ever-evolving opioid crisis.

We have outlined that narrative and discourse can have a significant impact on the way in which policies are prioritized [12]. We believe researchers have an important role in shaping future policy about substance use and mental health disorders by reframing ideas through knowledge translation, formation of values, creation of new knowledge and adding to the quality of public discourse and debate [73].

## Abbreviations

HAT: Heroin assisted treatment
OAT: Opioid agonist treatment
OUD: Opioid use disorder

## References

[1] Dhalla IA, Mamdani MM, Sivilotti ML, Kopp A, Qureshi O, Juurlink DN (2009). Prescribing of opioid analgesics and related mortality before and after the introduction of long-acting oxycodone. CMAJ.

[2] Kiepek N, Groom B, Toppozini D, Kakekagumick K, Muileboom J, Kelly L (2015). Evaluation of an inpatient medical withdrawal program in rural Ontario: a 1-year prospective study. Can J Rural Med.

[3] Lynas K (2013). Ontario pharmacists concerned about the risks arising from approval of generic OxyContin. Can Pharm J (Ott).

[4] Lynas K. Ontario police chiefs call on the federal government to keep generic OxyContin out of Canad. Canadian Pharmacist Journal. 2013;45 10.3821/145.5.cpj204 PMC3567579 23509559

[5] Standing committee on health. Office of the Speaker of the house of commons**.** Ottawa: Government of Canada; 2016.

[6] College of Physicians and Surgeons. In: CPSO methadone conference; 2015.

[7] Opioid Use and Related Adverse Events in Ontario. Ontario Drug Policy Research Network. http://odprn.ca/wp-content/uploads/2016/11/ODPRN-Opioid-Use-and-Related-Adverse-Events-Nov-2016.pdf.

[8] Astals M, Domingo-Salvany A, Buenaventura CC, Tato J, Vazquez JM, Martin-Santos R, Torrens M (2008). Impact of substance dependence and dual diagnosis on the quality of life of heroin users seeking treatment. Subst Use Misuse.

[9] Drake RE, Osher FC, Wallach MA (1991). Homelessness and dual diagnosis. Am Psychol.

[10] Drake RE, Brunette MF (1998). Complications of severe mental illness related to alcohol and drug use disorders. Recent Dev Alcohol.

[11] Galea S, Vlahov D (2002). Social determinants and the health of drug users: socioeconomic status, homelessness, and incarceration. Public Health Rep.

[12] Sumner A, Crichton J, Theobald S, Zulu E, Parkhurst J (2011). What shapes research impact on policy? Understanding research uptake in sexual and reproductive health policy processes in resource poor contexts. Health Res Policy Syst.

[13] Amato L, Davoli M, Perucci CA, Ferri M, Faggiano F, Mattick RP (2005). An overview of systematic reviews of the effectiveness of opiate maintenance therapies: available evidence to inform clinical practice and research. J Subst Abus Treat.

[14] Ball C (1991). The effectiveness of methadone maintenance treatment.

[15] Banta-Green CJ, Maynard C, Koepsell TD, Wells EA, Donovan DM (2009). Retention in methadone maintenance drug treatment for prescription-type opioid primary users compared to heroin users. Addiction.

[16] Bart G (2012). Maintenance medication for opiate addiction: the foundation of recovery. J Addict Dis.

[17] Mattick RP, Breen C, Kimber J, Davoli M. Methadone maintenance therapy versus no opioid replacement therapy for opioid dependence. Cochrane Database Syst Rev. 2009:CD002209. 10.1002/14651858.CD002209 12519570

[18] Herget G (2005). Methadone and buprenorphine added to the WHO list of essential medicines. HIV AIDS Policy Law Rev.

[19] Hopfer CJ, Khuri E, Crowley TJ, Hooks S (2002). Adolescent heroin use: a review of the descriptive and treatment literature. J Subst Abus Treat.

[20] Brugal MT, Domingo-Salvany A, Puig R, Barrio G, Garcia de Olalla P, de la Fuente L (2005). Evaluating the impact of methadone maintenance programmes on mortality due to overdose and aids in a cohort of heroin users in Spain. Addiction.

[21] Mattick RP, Breen C, Kimber J, Davoli M. Methadone maintenance therapy versus no opioid replacement therapy for opioid dependence. Cochrane Database Syst Rev. 2003:CD002209. 10.1002/14651858.CD002209 12804430

[22] Gowing L, Farrell M, Bornemann R, Ali R. Substitution treatment of injecting opioid users for prevention of HIV infection. Cochrane Database Syst Rev. 2004:CD004145. 10.1002/14651858.CD004145.pub2 15495080

[23] Marsch LA (1998). The efficacy of methadone maintenance interventions in reducing illicit opiate use, HIV risk behavior and criminality: a meta-analysis. Addiction.

[24] Novick DM, Richman BL, Friedman JM, Friedman JE, Fried C, Wilson JP, Townley A, Kreek MJ (1993). The medical status of methadone maintenance patients in treatment for 11-18 years. Drug Alcohol Depend.

[25] Glanz M, Klawansky S, McAullife W, Chalmers T (1997). Methadone vs. L-alpha-acetylmethadol (LAAM) in the treatment of opiate addiction. A meta-analysis of the randomized, controlled trials. Am J Addict.

[26] Fischer B, Kurdyak P, Goldner E, Tyndall M, Rehm J (2016). Treatment of prescription opioid disorders in Canada: looking at the 'other epidemic'?. Subst Abuse Treat Prev Policy.

[27] Eibl JK, Morin-Taus KA, Marsh DC (2016). Too much or never enough: a response to treatment of opioid disorders in Canada: looking at the 'other epidemic'. Subst Abuse Treat Prev Policy.

[28] Fischer B (2000). Prescriptions, power and politics: the turbulent history of methadone maintenance in Canada. J Public Health Policy.

[29] U.S. Department of Health and Human Services. https://www.surgeongeneral.gov/library/2016alcoholdrugshealth/index.html. 10.3109/15360288.2015.1037530 26095483

[30] Chiefs of Ontario.

[31] Patented medicine prices review board. In: Utilization of prescription opioids in Canada’s public drug plans, 2006/07 to 2012/13. Ottawa: Patented Medicine Prices Review Board; 2014.

[32] Russell C, Firestone M, Kelly L, Mushquash C, Fischer B (2016). Prescription opioid prescribing, use/misuse, harms and treatment among aboriginal people in Canada: a narrative review of available data and indicators. Rural Remote Health.

[33] Fayerman P. B.C. doctors first in Canada to face mandatory prescribing standards for opioids and other addictive drugs. In: Vancouver Sun. Vancouver; 2016. http://vancouversun.com/health/local-health/b-c-doctors-first-in-canada-to-face-mandatory-prescribing-standards-for-opioids-and-other-addictive-drugs.

[34] Dooling K, Rachlis M (2010). Vancouver's supervised injection facility challenges Canada's drug laws. CMAJ.

[35] Government of Canada. National Anti-drug Strategy. http://healthycanadians.gc.ca/anti-drug-antidrogue/index-eng.php.

[36] Schneider AI, H. Social construction of target populations: implications for politics and policy. Am Polit Sci Rev. 1993;

[37] Government of Ontario. Important Notice Regarding Change in Funding Status of Oxycodone Controlled Release Tablet. http://www.health.gov.on.ca/en/pro/programs/drugs/opdp_eo/notices/exec_office_odb_20120217.pdf.

[38] Cicero TJ, Ellis MS, Surratt HL (2012). Effect of abuse-deterrent formulation of OxyContin. N Engl J Med.

[39] Dart RC, Severtson SG, Bucher-Bartelson B (2015). Trends in opioid analgesic abuse and mortality in the United States. N Engl J Med.

[40] Opioid use increases after oxycodone crackdown. www.theglobeandmail.com/life/health-and-fitness/health/opioid-use-increases-after-oxycodone-crackdown/article19501813.

[41] Leece P, Orkin AM, Kahan M (2015). Tamper-resistant drugs cannot solve the opioid crisis. CMAJ.

[42] Dart RC, Surratt HL, Cicero TJ, Parrino MW, Severtson SG, Bucher-Bartelson B, Green JL (2015). Trends in opioid analgesic abuse and mortality in the United States. N Engl J Med.

[43] Wong A. Narcotics monitoring system (NMS) update. In: Ontario methadone prescribers conference. Toronto; 2015.

[44] Donovan K. Doctors exploit addicts to milk OHIP: task force. In: The Toronto star. Toronto; 2007.

[45] Fee cuts pushing Ontario doctors to close methadone clinics. http://www.theglobeandmail.com/news/national/fee-cuts-pushing-ontario-doctors-to-close-methadone-clinics/article27171502/.

[46] Boyle T. A methadone dispute and a system in trouble. In: Toronto Star. Toronto; 2014.

[47] Eibl JK, Gomes T, Martins D, Camacho X, Juurlink DN, Mamdani MM, Dhalla IA, Marsh DC (2015). Evaluating the effectiveness of first-time methadone maintenance therapy across northern, rural, and urban regions of Ontario, Canada. J Addict Med.

[48] Brands JB, Brands B, Marsh DM (2000). The expansion of methadone prescribing in Ontario, 1996–1998. Addict Res.

[49] Kiepek N, Hancock L, Toppozini D, Cromarty H, Morgan A, Kelly L (2012). Facilitating medical withdrawal from opiates in rural Ontario. Rural Remote Health.

[50] MMT practice task force report. http://www.health.gov.on.ca/en/common/ministry/publications/reports/methadone_taskforce/methadone_taskforce.pdf.

[51] Astals M, Diaz L, Domingo-Salvany A, Martin-Santos R, Bulbena A, Torrens M (2009). Impact of co-occurring psychiatric disorders on retention in a methadone maintenance program: an 18-month follow-up study. Int J Environ Res Public Health.

[52] Canadian Insitute for Health Information. Opioid-Related Harms in Canada. https://www.cihi.ca/sites/default/files/document/opioid-harms-chart-book-en.pdf.

[53] International guidelines for estimating the costs of substance abuse. http://citeseerx.ist.psu.edu/viewdoc/download?doi=10.1.1.1016.5362&rep=rep1&type=pdf.

[54] Hansen RN, Oster G, Edelsberg J, Woody GE, Sullivan SD (2011). Economic costs of nonmedical use of prescription opioids. Clin J Pain.

[55] Kingston Frontenac and Addington Public Health Unit. https://public.tableau.com/profile/kflaphi#!/vizhome/OntarioOpioidSurveillanceMonitor/ACESEDVisits.

[56] World Health Organization. Principles of Drug Dependence Treatment. http://www.who.int/substance_abuse/publications/principles_drug_dependence_treatment.pdf?ua=1.

[57] Keen L, Khan M, Clifford L, Harrell PT, Latimer WW (2014). Injection and non-injection drug use and infectious disease in Baltimore City: differences by race. Addict Behav.

[58] CAMH: Do you know? Opioids. Health. CfAaM ed.; 2013.

[59] Gomes T, Mamdani MM, Dhalla IA, Cornish S, Paterson JM, Juurlink DN (2014). The burden of premature opioid-related mortality. Addiction.

[60] Patients First: Action Plan for Health Care. http://www.health.gov.on.ca/en/ms/ecfa/healthy_change/docs/rep_patientsfirst.pdf.

[61] Powell B (2016). Toronto council approves 3 supervised injection sites. Toronto Star.

[62] City seeks federal nod to open supervised injection sites. https://www.thestar.com/news/city_hall/2016/11/30/city-seeks-federal-nod-to-open-supervised-injection-sites.html.

[63] Health minister approves 3 supervised drug consumption sites in Montreal. http://www.cbc.ca/news/politics/safe-consumption-site-montreal-1.3969258.

[64] Rush (2016). NE LHIN addiction services review.

[65] Hausser D, Kubler D, Dubois-Arber F (1999). Characteristics of heroin and cocaine users unknown to treatment agencies. Results from the Swiss hidden population study. Soz Praventivmed.

[66] The last thing I would have expected. http://www.theglobeandmail.com/news/british-columbia/north-van-couple-lost-tofentanyl/article32933591/.

[67] Brend Y. North Vancouver couple's sudden death shocks family. In: CBC. Vancouver: British Columbia; 2015.

[68] Kerr T, Stoltz JA, Tyndall M, Li K, Zhang R, Montaner J, Wood E (2006). Impact of a medically supervised safer injection facility on community drug use patterns: a before and after study. BMJ.

[69] Wood E, Tyndall MW, Montaner JS, Kerr T (2006). Summary of findings from the evaluation of a pilot medically supervised safer injecting facility. CMAJ.

[70] Zaric GS, Bayoumi AM, Brandeau ML, Owens DK (2008). The cost-effectiveness of counseling strategies to improve adherence to highly active antiretroviral therapy among men who have sex with men. Med Decis Mak.

[71] Gartry CC, Oviedo-Joekes E, Laliberte N, Schechter MT (2009). NAOMI: the trials and tribulations of implementing a heroin assisted treatment study in North America. Harm Reduct J.

[72] Vacouver Coastal Health. Supervised Injection Sites. http://www.vch.ca/public-health/harm-reduction/supervised-injection-sites.

[73] Davies H, Nutley S, Walter I. A background discussion paper for assessing the impact of social science research: conceptual, methodological and practical issues. In: ESRC Symposium on Assessing Non-Academic Impact of Research. St. Andrew University; 2005.

